# Nanodiamond-supported silver nanoparticles as potent and safe antibacterial agents

**DOI:** 10.1038/s41598-019-49675-z

**Published:** 2019-09-11

**Authors:** Be-Ming Chang, Lei Pan, Hsin-Hung Lin, Huan-Cheng Chang

**Affiliations:** 1 Institute of Atomic and Molecular Sciences, Academia Sinica, Taipei, 106 Taiwan; 2 Taiwan International Graduate Program, Molecular Science and Technology, Academia Sinica, Taipei, 115 Taiwan; 3 Department of Chemistry, National Tsing Hua University, Hsinchu, 300 Taiwan; 4 Department of Chemical Engineering, National Taiwan University of Science and Technology, Taipei, 106 Taiwan

**Keywords:** Lifestyle modification, Nanoparticles, Drug delivery

## Abstract

Since its discovery nearly a century ago, antibiotics has been one of the most effective methods in treating infectious diseases and limiting pathogen spread. However, pathogens often build up antibiotic resistance over time, leading to serious failure of the treatment. Silver nanoparticle (AgNP) is an appealing alternative, but successful treatment of the bacterial infection requires a plentiful supply of AgNP, which can negatively impact human health if people are excessively exposed to the particles. Here, we present a method to overcome this challenge by synthesizing nanodiamond-supported AgNP noncovalently conjugated with albumin molecules to achieve enhanced antibacterial activity and strengthened biocompatibility. Using *Escherichia coli* as a model bacterium, we found that the albumin-conjugated silver-diamond nanohybrids showed a long-term bactericidal effect after 36 days of the treatment at the AgNP concentration of 250 µg mL^−1^. Moreover, the toxicity of the nanohybrids to human cells (including human fibroblasts, lung adenocarcinoma epithelial cells, and breast adenocarcinoma cells) is low even at the particle concentration of 500 µg mL^−1^. The method provides a general and practical solution to the concerns of bacterial resistance against AgNP and issues associated with the size, shape, aggregation, and toxicity of AgNP are largely resolved. Finally, we demonstrate that the nanohybrids can be readily incorporated into natural polysaccharides (such as guar gum) to form three-in-one hydrogels, showing promising applications in nanomedicine.

## Introduction

Infectious disease is one of the serious threats to global health^1^. Microbial infection causes dangerous diseases in human such as cholera, diphtheria, pneumonia, and many more^2^. Inflammatory reactions such as fever often occur during serious medical operations like surgery or transplant replacement. Patients’ weakened immune systems have difficulties in defending against deadly infections even from a tiny scrape on the skin. The high demand to treat infections eventually led to the discovery of antibiotics such as Penicillin, which have saved billions of lives since its discovery in 1928 and have played a central role in pharmaceuticals and hospitals^3^. However, injudicious uses of antibiotics over the past few decades have made infections harder to treat and antibiotic resistance is now a major threat to public health worldwide^4–7^.

For centuries, silver has been recognized as a natural antibiotic material, able to cure various diseases^8^. Recently, nanoscale silver is emerging as a new generation of the treatments for their high antimicrobial efficiency and distinct modes of action from that of conventional antibiotics^9,10^. These silver nanoparticles (AgNPs) are typically 20 nm in diameter and have large specific surface areas, allowing them to form good contact with bacteria. Moreover, they are capable of attacking a wide range of targets on plasma membranes as well as in cells, making microorganisms difficult to develop resistance^11^. Despite the promise of using AgNP to fight against multidrug-resistant pathogens or “superbugs”, there are increasing concerns about the development of bacterial resistance to the nanoparticles^12,13^. Panáček *et al*.^13^ has recently reported that the resistance of *Escherichia coli* (*E. coli*) to AgNP is resulted from the aggregation of the nanoparticles in medium, triggered by adhesive flagellum proteins released from the bacteria. To avoid the resistance development, the simplest and most general solution is by using a high level of AgNP (such as 10× that of the minimal inhibitory concentration, MIC) to ensure rapid and long-term bactericidal effects. The application, however, is hampered by the easy aggregation of the metal nanoparticles in biological medium and their toxicity in human cells^14–16^. Therefore, there is an urgent need to develop AgNP-based antimicrobial agents with high activity but low human toxicity.

An ideal antimicrobial agent should be selectively toxic, soluble and stable in body fluids, nonallergenic, and free of resistance development^17^. Hybrid AgNP is a promising candidate to meet these requirements^18^. Among various nanoparticles that may serve as the partner of AgNP in the hybrid, nanodiamond (ND) stands out for its intrinsic biocompatibility and fluorescing capability, and have found applications in diverse research areas as drug delivery vehicles, bioimaging agents, and biosensing devices^19,20^. Hemocompatibility studies have shown that acid-washed ND, after intravenous administration into mice, induces negligible inflammation and toxicity of the treated animals^21^. Moreover, ND surface-functionalized with carboxyl groups by acid washes can be covalently conjugated with cationic polymers and then other types of nanoparticles such as citrate-capped gold nanorods to form hybrids or composites through electrostatic interactions^22^. These nanohybrids, after additional coating with bovine serum albumin (BSA) by physical adsorption, exhibit excellent dispersibility and stability in physiological medium such as phosphate-buffered saline (PBS) for weeks^23^.

Here, we report a method of synthesizing Ag-ND@BSA hybrids and demonstrate their promising use in nanomedicine as potent and safe antibacterial agents. The nanohybrids synthesized have a size in the range of 100–200 nm, showing an excellent colloidal stability in bacterial culture. Additionally, they are small enough to make good contacts and close interactions with bacteria like *E. coli* (typical 1–2 μm in diameter)^24–26^, but are too large to penetrate membrane bilayers of human cells (typical 10–100 μm in diameter) to cause significant cytotoxic effects. Furthermore, thanks to the exceptional biocompatibility and mechanical robustness of the ND support, these nanohybrids can be facilely incorporated into a wide range medical supplies and appliances as well as personal care products.

## Results

**Figure 1:**
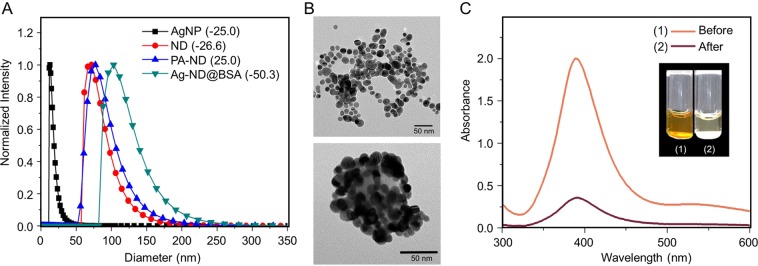
Characterization of AgNP and Ag-ND@BSA. (**A**) Dynamic light scattering of citrate-capped AgNP, ND, PA-ND, and Ag-ND@BSA suspended in water. Values in parentheses are zeta potentials (in unit of mV) of the corresponding nanoparticles. (**B**) Transmission electron micrographs of AgNP (top) and Ag-ND@BSA (bottom). (**C**) Quantification of AgNP attached to ND in Ag-ND@BSA by UV-Vis absorption spectroscopy. Inset: Photographs of an AgNP suspension (1) and a supernatant of the AgNP suspension (2) after adding ND to form the Ag-ND hybrids precipitated by centrifugation.

The ND particles used in this work were monocrystalline diamond powders with a nominal diameter of 100 nm. They were first oxidized in air at 450 °C to remove graphitic surface structure and then thoroughly washed in concentrated acid mixtures (H_2_SO_4_:HNO_3_ = 3:1) at 90 °C to eliminate any possible metallic components and, concurrently, functionalize their surface with carboxyl groups for ensuing conjugation with poly-L-arginine (PA) through carbodiimide chemistry (Supplementary Scheme S1)^22^. Figure 1A shows the size distributions and surface polarities of acid-washed ND and PA-conjugated ND characterized by dynamic light scattering and zeta potential analysis. With a zeta potential of +25 mV, the PA-conjugated ND was able to bind strongly with citrate-capped AgNP (zeta potential of −25 mV and diameter of 17 ± 6 nm) synthesized by using AgNO_3_ and a standard chemical reduction method^27^. Transmission electron microscopy confirmed the attachment of the citrate-capped AgNP to the PA-ND with a surface coverage as high as 80% in cluster form (Fig. 1B). To enhance the bactericidal ability^28^, the assembled AgNP-ND (or Ag-ND in short) was additionally oxidized in water by oxygen bubbling prior to coating with BSA by physical adsorption^23^. The end product (denoted as Ag-ND@BSA) had a zeta potential of −50 mV as BSA is an anionic protein with an isoelectric point around 4.7. The final size of the nanohybrids was 120 ± 30 nm, compared with 84 ± 22 nm of acid-washed ND before the surface modification.

An important quantity to determine in this study is the absolute amount of AgNP attached to each ND particle in Ag-ND@BSA. This was achievable by using ultraviolet-visible absorption spectroscopy for the plasmonic band of AgNP at 405 nm (Fig. 1C). We first determined the yield of AgNP produced by the chemical reduction method using ultra-centrifugation to collect the nanoparticles and measure their dry weight (Supplementary Fig. S1), followed by a quantification for the amount of AgNP anchored on the ND surface by measuring the absorption intensity changes of unbound AgNP at 405 nm before and after addition of PA-ND into the AgNP solution with a known concentration (in unit of μg mL^−1^). For 17-nm AgNP attached to 100-nm ND, we obtained a loading capacity of 1.0 ± 0.2 g g^−1^, or ~80 AgNP per ND, assuming a spherical shape for both nanomaterials.

Two representative bacteria, *E. coli* (K12 MG1655) and *Staphylococcus aureus* (*S. aureus*), were used to test the antimicrobial activities of Ag-ND@BSA. The former is a Gram-negative bacterium and the latter is a Gram-positive bacterium, both of which can cause many diseases and a variety of infections^29–31^. We began the testing with AgNP to examine how they aggregated in culture medium and how the particle aggregation affected their antimicrobial activities for *E. coli*. Particles coated with BSA and lactalbumin (LA) served as the control experiments. Similar to BSA (66 kDa), LA is an anionic protein but with a smaller molecular weight (14 kDa) and therefore can offer a better coverage than BSA for AgNP of ~20 nm in diameter. Our results showed that AgNP alone precipitated rapidly after being added to the Luria-Bertani (LB) broth, which is a nutrition-rich medium for the growth of bacteria (Supplementary Fig. S2). In contrast, no significant aggregation and precipitation occurred for AgNP@LA in the same medium at the concentration higher than 1 mg mL^−1^, signifying the importance of LA coating.

**Figure 2:**
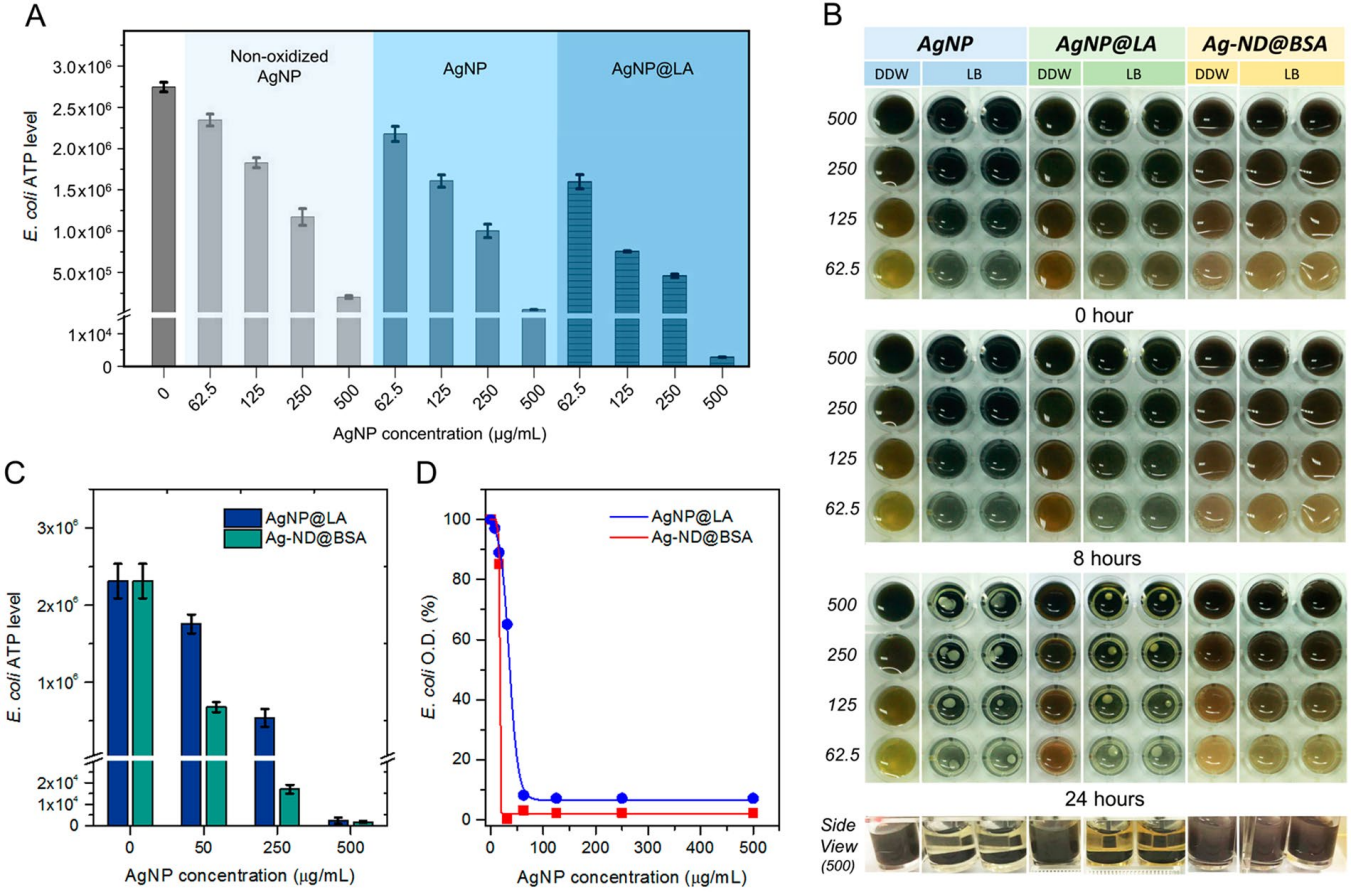
Antibacterial activities of AgNP, AgNP@LA, and Ag-ND@BSA. (**A**) Antimicrobial tests of non-oxidized AgNP, AgNP, and AgNP@LA against *E. coli*. (**B**) Aggregation and precipitation of three different types of particles in LB broth deposited on microplates. The concentration of these particles decreases from 500 µg mL^−1^ to 62.5 µg mL^−1^ (1st to 4th row at each time point). The particles in the first column of each sample were dispersed in distilled deionized water (DDW), serving as the negative control. The side view shows the precipitation of AgNP and AgNP@LA at 500 µg mL^−1^ after the sample preparation for 24 h. (**C**) Concentration-dependent measurements for the antibacterial activities of AgNP@LA and Ag-ND@BSA at 50, 250, and 500 µg mL^−1^. (**D**) MIC measurements of AgNP@LA and Ag-ND@BSA. The measurements were conducted with a spectrophotometer for the O.D. of the *E. coli* suspensions at 600 nm.

To examine the bactericidal properties of AgNP and AgNP@LA for *E. coli*, a microbial cell viability assay (BacTiter-Glo) was applied to quantify the levels of adenosine triphosphate (ATP) in the bacteria. The assay is based on the measurement for the luciferase activity, thus providing a real-time monitoring capability and a broad dynamic range. No antibacterial effect was found during the control treatment with BSA and LA only (Supplementary Fig. S3), whereas the viability of *E. coli* was compromised in the presence of AgNP with the bacterial ATP level dropping by more than one order of magnitude at the particle concentration of 500 µg mL^−1^ (Fig. 2A). Further reduction of the ATP levels by 5–10 times was achievable if the same AgNP particles were stabilized by LA. The finding is in line with the suggestion that preventing AgNP from aggregation and subsequent precipitation is crucial to attaining a high bactericidal effect^13^.

Next, we compared side-by-side the bactericidal effect between AgNP@LA and Ag-ND@BSA by performing dose-dependent measurements. A colloidal stability testing of these two types of particles in LB over 24 h indicated that Ag-ND@BSA outperformed AgNP@LA in terms of its long-term dispersibility within the concentration range of 62.5–500 µg mL^−1^ (Fig. 2B). The addition of AgNP@LA to the LB broth at a concentration of 50 µg mL^−1^ resulted in a reduction of the bacterial ATP level by only 30% (2.3 × 10^6^ to 1.8 × 10^6^), whereas the addition of Ag-ND@BSA diminished the bacterial activity by a much larger margin (2.3 × 10^6^ to 6.8 × 10^5^) at the same effective AgNP concentration (Fig. 2C). The ATP level was further reduced by 40-fold (1.7 × 10^4^) as the particle concentration increased to 250 µg mL^−1^ and was diminished almost completely (1.7 × 10^3^) at 500 µg mL^−1^. The superior performance of Ag-ND@BSA was also reflected in its minimum inhibitory concentration (MIC) value, defined as the lowest concentration of an agent that inhibits visible growth of a microbe after overnight incubation^32^. We determined a MIC value of 63 µg mL^−1^ and 15 µg mL^−1^ for AgNP@LA and Ag-ND@BSA, respectively, by optical density (O.D.) measurement after incubation of the bacteria with the particles for 18 h (Fig. 2D), showing a 4-fold strengthened bactericidal effect thanks to the support by ND. The potency of Ag-ND@BSA is ~96%, compared with ~92% of AgNP@LA.

Apart from MIC, the minimum bactericidal concentration (MBC) is another criterion useful to classify the tested microorganism for its susceptibility or resistance to the tested drugs. The parameter reveals the lowest concentration of a bactericide that results in microbial death. We determined the MBC by sub-culturing the particle-treated *E. coli* for another 18 h on the LB agar plates, observed the growth of bacterial colonies, and found that the MBC of the treatment was 63 µg mL^−1^ and 31 µg mL^−1^ for AgNP@LA and Ag-ND@BSA, respectively. Increasing the doses to 250 µg mL^−1^ clearly ensured complete protection against bacteria (Supplementary Fig. S4). The higher potency of Ag-ND@BSA than AgNP@LA was attributed in part to a local concentration effect, where many AgNP particles could interact simultaneously with the bacterium in a tiny area (e.g. 100 × 100 nm^2^) as soon as Ag-ND@BSA was in contact with the microorganism.

**Figure 3:**
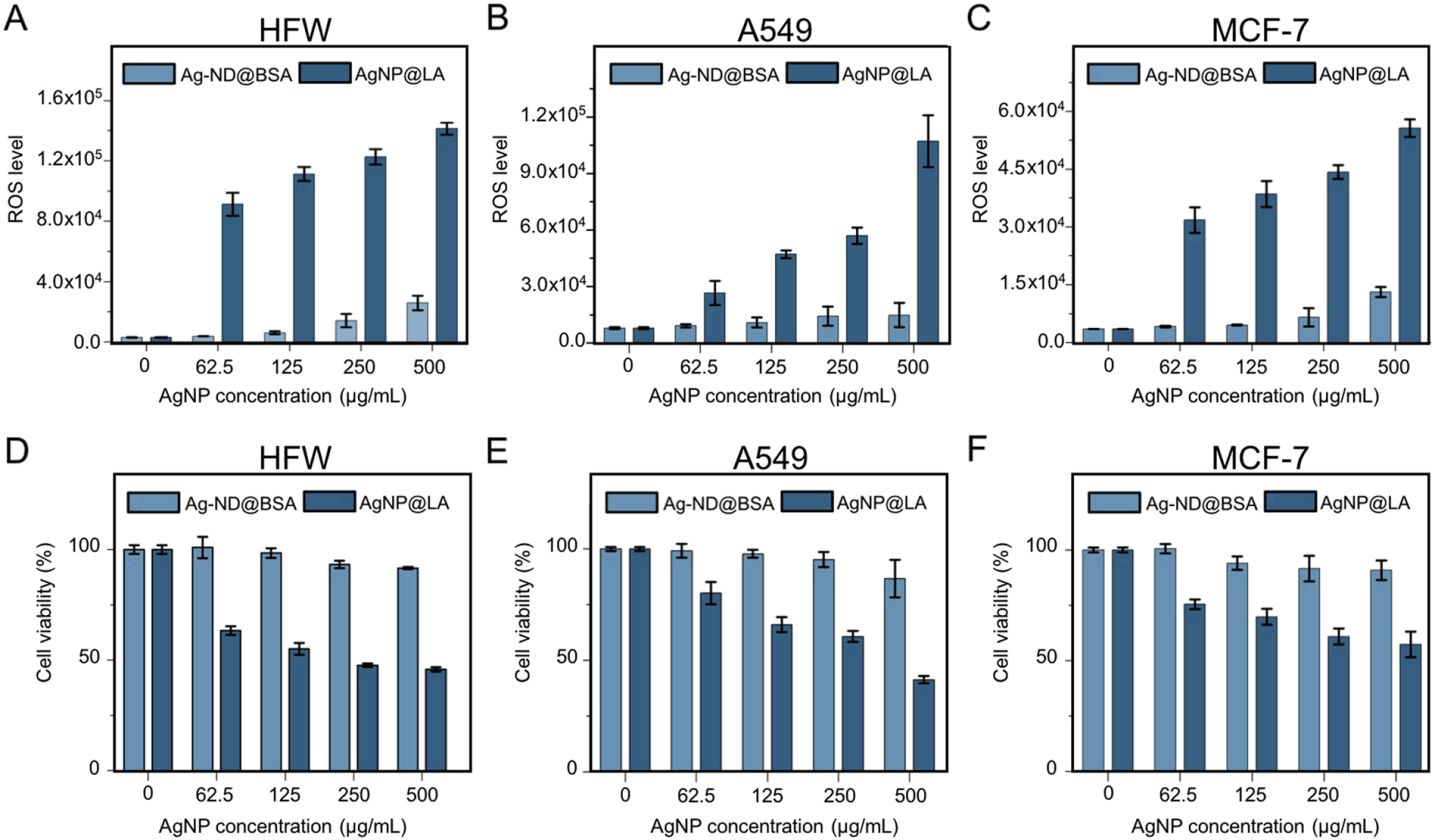
Cytotoxicity assessments of AgNP@LA and Ag-ND@BSA. (**A–C**) ROS and (**D–F**) viability assays of HFW, A549, and MCF-7 cells incubated with AgNP@LA or Ag-ND@BSA. The ROS levels were measured with the ROS-Glo H_2_O_2_ assay and the cell viability measurements were conducted with the cell counting kit-8 assay.

While Ag-ND@BSA shows sustained bactericidal effects, the utility of the treatment is compromised if the nanohybrids are toxic to human cells at high concentrations such as 250 µg mL^−1^. To address this issue, three cell lines including human fibroblasts (HFW), lung adenocarcinoma epithelial cells (A549), and breast adenocarcinoma cells (MCF-7) were used for cytotoxicity assessment. We started the measurement with the ROS-Glo H_2_O_2_ assay for the generation of intracellular reactive oxygen species (ROS) that are involved in apoptosis signaling^33^. The effects of both AgNP@LA and Ag-ND@BSA on the ROS levels of the cells were then monitored over the particle concentration of 62.5–500 µg mL^−1^ for 1 h. As shown in Fig. 3A–C, AgNP@LA induced significantly higher ROS levels than Ag-ND@BSA for all three cell types. The result is consistent with the cell viability assessment using the cell counting kit-8 assay (CCK-8), which measured the activity of dehydrogenases in living cells. More than 80% of the cells were still viable even in medium containing 500 µg mL^−1^ Ag- ND@BSA after 24-h incubation before cell proliferation occurred. Remarkably, the percentages of viable cells could remain higher than 70% under the same treatment even after incubation for 48 and 72 h (Supplementary Fig. S5). In contrast, the cell viability dropped to be less than 50% due to the lack of the ND support (Fig. 3D–F). With a half cytotoxic concentration (CC50) in the range of 250–500 µg mL^−1^ for the three human cell types, we estimated a selectivity index (SI) of CC(50)/IC(50) = 10–20 for the AgNP@LA particles. The SI value of Ag-ND@BSA, however, could not be determined due to the exceptional low cytotoxicity of the nanohybrids, with a CC50 much greater than 500 µg mL^−1^.

**Figure 4:**
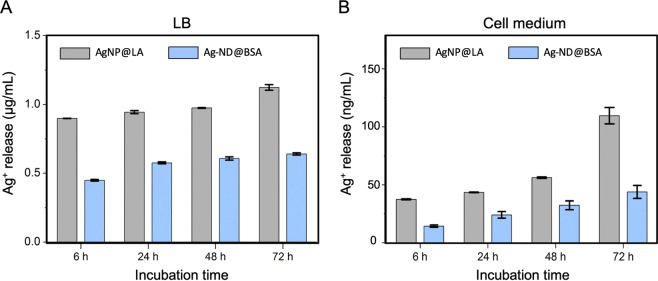
Time-dependent quantification of silver ions released from AgNP@LA and Ag-ND@BSA in culture medium: (**A**) LB broth and (**B**) cell medium. The experiments were conducted at 500 µg mL^−1^ AgNP@LA and Ag-ND@BSA for 6, 24, 48, and 72 h at 37 °C. Notice the 10x difference in the vertical scales between graphs in panels A and B.

For practical use, the Ag-ND@BSA hybrids must be able to elute a sufficient amount of silver ions to efficiently and persistently kill bacteria over an extended period of time. These ions can form through the oxidative reaction between silver atoms on the AgNP surface with ambient O_2_ dissolved in solutions^34^. Inductively coupled plasma mass spectrometry served well to quantify the Ag^+^ release^35^. Figure 4 shows the results of the quantification for AgNP@LA and Ag-ND@BSA in both LB broth and cell medium at the particle concentration of 500 µg mL^−1^ over an incubation time of 72 h at 37 °C. The Ag^+^ concentrations increased steadily with time and, interestingly, exhibited a 10 × higher level in the bacteria growth medium than in the mammalian cell medium for both AgNP@LA and Ag-ND@BSA. More importantly, there were nearly twice more Ag^+^ ions released from AgNP@LA than from Ag-ND@BSA, which could be the major cause for the higher mortality of human cells in the AgNP@LA treatment (Fig. 3).

**Figure 5:**
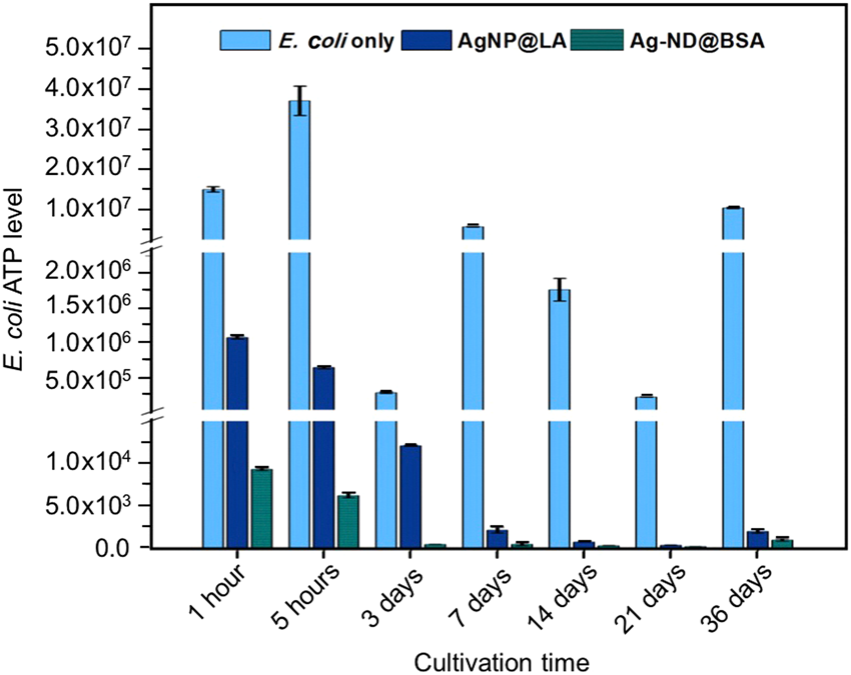
Time-dependent bactericidal activities of AgNP@LA and Ag-ND@BSA. The measurements were conducted at 250 µg mL^−1^ in LB broth for *E. coli* over 36 days with the BacTiter-Glo microbial cell assay.

To explore the feasibility of clinical applications, we examined the antibacterial effect of the nanohybrids at 250 µg mL^−1^ in LB broth starting at 1 h and tracked the results over a month. Within the first hour, the Ag-ND@BSA particles inhibited the explosive growth of *E. coli* with an ATP activity level of 9.3 × 10^3^, lower than that (1.5 × 10^7^) of *E. coli* alone by more than 3 orders of magnitude (Fig. 5). The AgNP@LA treatment, in contrast, left around 7% bacteria active (with an ATP level of 1.1 × 10^6^), which could cause hidden threats to patients in clinical applications. At the 5-h incubation time point, the Ag-ND@BSA treatment showed a further suppression of the ATP activity to 6.2 × 10^3^ and the bacteria were almost eradicated on day 3. Such a distinctive outcome lasted longer than a month, constantly keeping the bacterial activity below 0.01% of the control level, demonstrating that the Ag-ND@BSA particle may serve well as an efficient sustained reservoir for slow release of silver ions to achieve long-term antibacterial effects.

## Discussion

**Figure 6:**
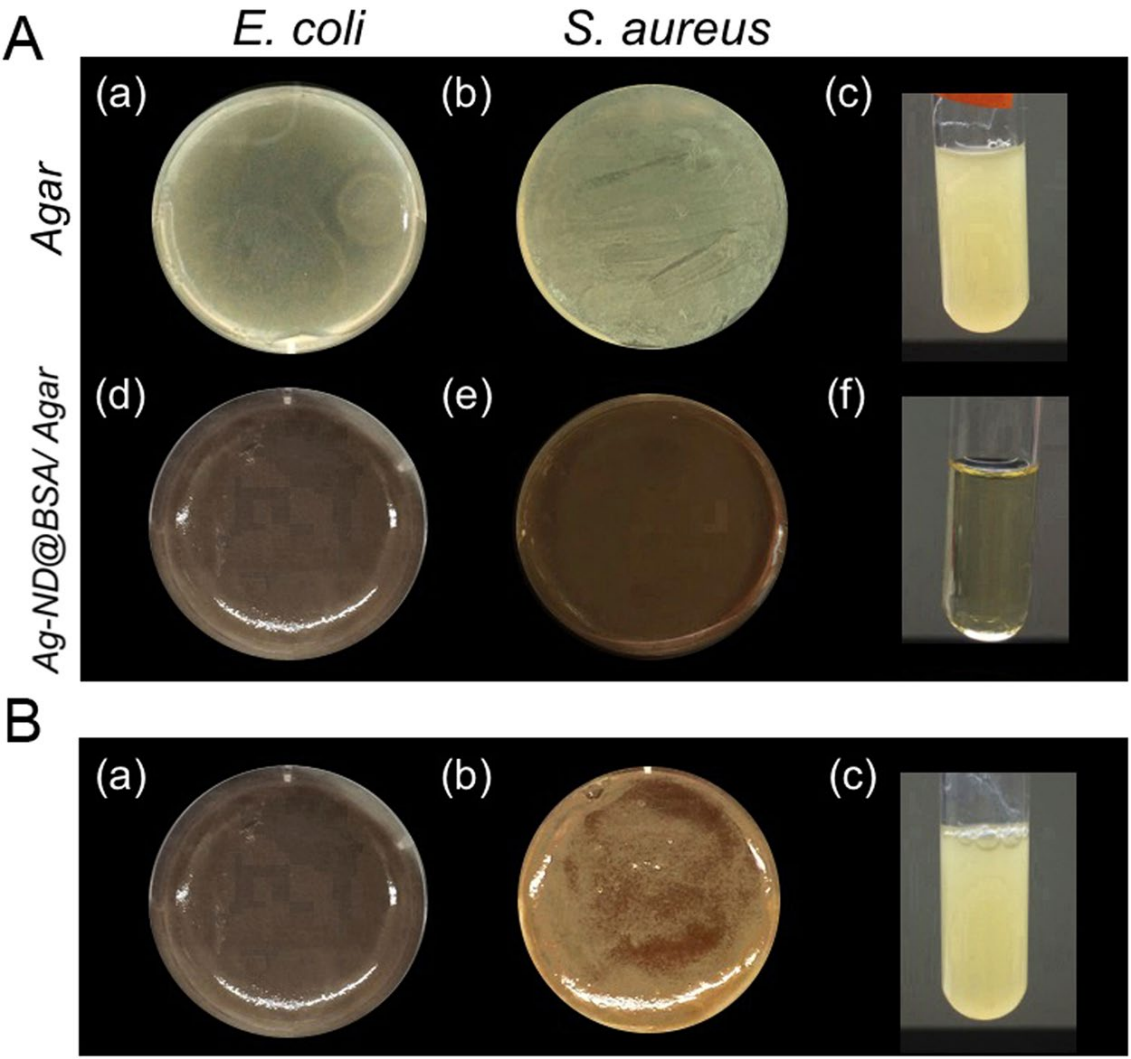
Bactericidal activities of Ag-ND@BSA-embedded agar plates. (**A**) Bacterial growth inhibition testing of Ag-ND@BSA embedded in agar plates for Gram-positive and Gram-negative bacteria. *E. coli* and *S. aureus* were cultured separately on agar (a, b) and Ag-ND@BSA/agar (d, e). Streaked bacteria from (a) and (d) were sub-cultured in fresh LB broth for another 18 h, showing bacterial growth in (c) and (f), respectively. (**B**) Bacterial growth inhibition testing of Ag-ND embedded in agar plates for *E. coli*. In contrast to the result of Ag-ND@BSA/agar (a), colonies clearly formed in the regions containing Ag-ND aggregates (b), showing the failure of preventing bacteria from proliferation. Bacterial growth appeared when streaked bacteria from (b) were sub-cultured in fresh LB broth for another 18 h in (c). The LB-supplemented agars in both experiments were mixed with 250 µg mL^−1^ Ag-ND@BSA or Ag-ND to form plates.

The development of AgNP nanohybrids with potent bactericidal activity and enhanced biosafety provides a rich toolbox for treating diseases. To illustrate the applications, we synthesized Ag-ND@BSA-incorporated agar plate to form a three-in-one hydrogel^36,37^. Both *E. coli* (Gram-negative) and *S. aureus* (Gram-positive) were employed to evaluate the utility of such agar plates as bactericide-incorporated/coated medical devices. As shown by the photographs in Fig. 6A, the Ag-ND@BSA-embedded agars could effectively inhibit both *E. coli* and *S. aureus* growth, clearly proving their ability to prevent device-associated infections caused by microbial attachment. In contrast, if the agar plates were embedded with Ag-ND without BSA coating, the nanoparticles facilely aggregated and some of the bacteria could still grow prosperously on the Ag- ND-embedded agar due to inhomogeneous distribution of the aggregated particles in the medium, leaving some room for the microorganisms to survive and proliferate (Fig. 6B).

**Figure 7:**
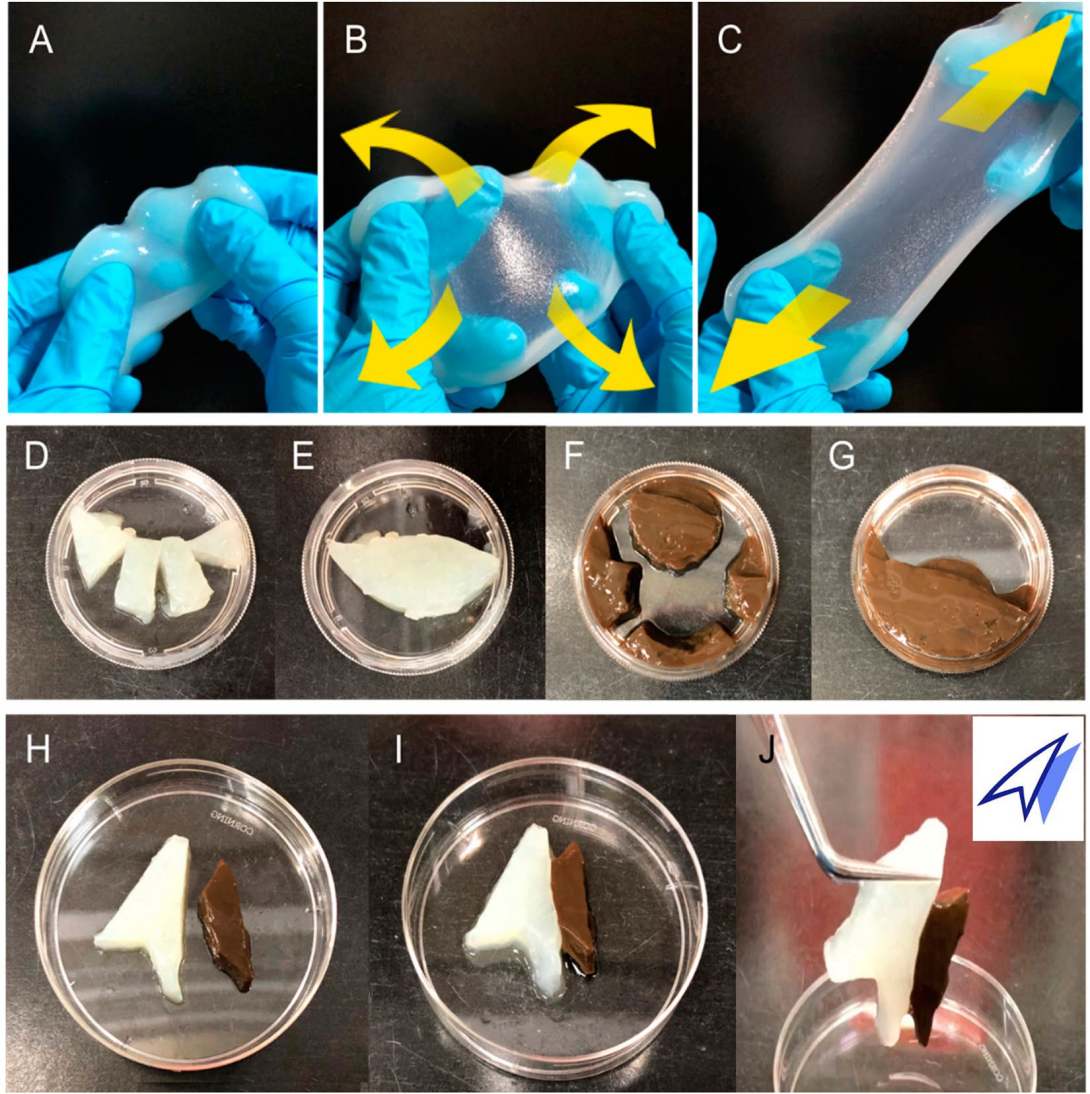
Physical properties of ND@BSA and Ag-ND@BSA-encrusted hydrogels. (**A–C**) Photographs showing the homogeneity and flexibility of a guar-based hydrogel embedded with ND@BSA. The hydrogel maintained its integrity when suffering tensile stress or strong elongation along the directions indicated by the yellow arrows in (**B**,**C**). (**D**–**J**) Photographs showing the shaping and reassembling abilities of ND@BSA- and Ag-ND@BSA-embedded hydrogels. The 2-in-1 or 3-in-1 hydrogels were first cut into four segments (**D,F**) and then reassembled into one piece through physical contacts as shown in (**E,G**), respectively. Different types of hydrogels could also be assembled together in a specific shape or arrangement (**H,I**). Neither deformation and nor rupture occurred as the self-healed hydrogel was exposed to an external force (**J**).

The experiments with hydrogels composed of AgNP, ND, and agar plainly illustrated the potent antibacterial ability of Ag-ND@BSA even in nutrient-rich environments. An immediate application of the technique is to fabricate three-in-one devices with a natural polysaccharide-based hydrogel (such as guar gum) incorporated with Ag-ND@BSA. These devices could easily form by crosslinking of monomers in the particle-dispersed pre-gel solution with chemicals such as tetraborate^38^. They have the merits of potent bactericidal property, high water content, as well as the capability of extreme stretching and shaping for wound dressing applications (Fig. 7A–C). Moreover, the Ag-ND@BSA-embedded hydrogels were able to self-heal into an integral part through physical contacts. Neither deformation and nor rupture occurred as the self-healed hydrogel was exposed to external forces, as illustrated in Fig. 7D–J. The feasibility of fabricating these AgNP-incorporating hydrogels highlights the potential use of these nanohybrids for coating on or incorporating in a wide range of medical supplies and appliances^39,40^.

**Figure 8:**
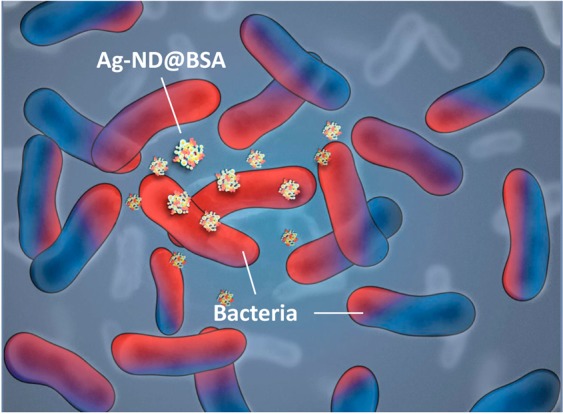
Illustration of how Ag-ND@BSA interacts with *E. coli*. Bacteria highly affected by the Ag-ND@BSA treatment are indicated in red.

To conclude, we have demonstrated that albumin-conjugated ND-supported AgNP particles have potent antimicrobial activity on both Gram-negative and Gram-positive bacteria but with low toxicity on human fibroblast and other cell types. These particles, having sizes in the range of 100‒200 nm, can interact intimately with bacteria through noncovalent conjugation (Fig. 8). Moreover, they act as silver ion reservoirs with enhanced antibacterial activity and reduced bacterial resistance, which make them appealing alternatives to antibiotics. These nanohybrids can be easily embedded in natural polysaccharide-based hydrogels to form bactericidal wound dressings for effective and sustainable antimicrobial treatments. As infections continue to pose severe threats to global health, the ND-supported AgNP-incorporated devices offer a possible solution to commonly encountered medical supplies and devices-related infection issues. Either with or without the addition of other drugs to improve their medical effects, these multifunctional three-in-one hydrogels promise professional pattern development to produce customized products. They are readily applicable for practical clinical use in hospitals.

## Methods

### Materials and chemicals

𝛼-Lactalbumin (LA), bovine serum albumin (BSA), N-(3-dimethylaminopropyl)-N′-ethylcarbodiimide hydrochloride (EDC), N-hydroxysuccinimide (NHS), poly-L-arginine hydrochloride (PA), silver nitrate (AgNO_3_), sodium borohydrate (NaBH_4_), and guar gum were obtained from Sigma-Aldrich, sodium citrate was from J. T. Baker, sodium tetraborate was from Fluka, Luria-Bertani (LB) broth and Dulbecco’s modified Eagle’s medium (DMEM) were from ThermoFisher Scientific, agar was from Difco, and synthetic diamond powders (Micron + MDA) were from Element Six.

### AgNP

Sodium citrate was prepared in distilled deionized water (DDW) with a concentration of 0.7 mM, which was then purged thoroughly with nitrogen gas for 1 h to remove dissolved molecular oxygen. After sealing the reaction bottle with septa to keep the supplied nitrogen, 0.1 M AgNO_3_ (100 μL) was injected into the citrate solution (100 mL), followed by drop-by-drop injection of 5 mg mL^−1^ NaBH_4_ in methanol (100 μL)^41^. The color of the solution changed visibly from transparent to yellow or dark brown after overnight reaction. A UV-Vis photospectrometer (U-3310, Hitachi) characterized the formation of AgNP particles at the wavelength of 405 nm. The final products were collected by ultracentrifugation (30,000 rpm, 1 h), dried in air, and then weighed to determine the yields. A 10-fold more concentrated product could be generated by increasing the concentrations of the individual reagents in proportion (Supplementary Fig. S1).

### ND

Diamond powders with a nominal diameter of 100 nm were oxidized in air at 450 °C to remove graphitic surface structure. They were then treated in concentrated acid mixtures (H_2_SO_4_:HNO_3_ = 3:1) at 90 °C to eliminate any possible metallic components and, concurrently, functionalize their surfaces with carboxyl groups. After thorough dispersion in acidic water (pH 4) by sonication for 15 min and mixing with N-(3-dimethylaminopropyl)-N′-ethylcarbodiimide hydrochloride (EDC, 20 mg) and N-hydroxysuccinimide (NHS, 20 mg) to activate surface –COOH groups for 30 min, the acid-treated ND particles (10 mg) were washed three times with DDW to remove unreacted reagents and then redispersed in basic solution (pH 8). PA (20 mg) was finally added to react with the amine-reactive ND for 6 h to form PA-conjugated ND.

### Ag-ND hybrids

PA-ND was thoroughly washed with DDW to remove unbound PA and subsequently mixed with the citrate-capped AgNP (20 mg) for 2 h. The amount of AgNP attached to PA-ND was determined by measuring the absorbance changes of unbound AgNP at 405 nm before and after addition of PA-ND into the AgNP solution with known concentration (in unit of mg mL^−1^). Specifically, the latter measurement was performed after centrifugal removal of ND to avoid the light scattering issue caused by the nanoparticles. To prepare AgNP@LA and Ag-ND@BSA, suspensions containing AgNP and PA-ND were first bubbled with oxygen gas for at least 30 min for surface oxidation of silver, followed by coating of the products with LA (LA:AgNP ~ 8:1 in weight ratio) and BSA (BSA:PA-ND ~1:1 in weight ratio). Excessive protein molecules were washed away with DDW. Hydrodynamic sizes and zeta potentials of the citrate-capped AgNP, acid-washed ND, PA-coated ND, AgNP@LA, and Ag-ND@BSA particles were measured with a particle size and zeta potential analyzer (DelsaNano C, Beckman Coulter). To examine the morphologies of AgNP and Ag-ND@BSA, the samples were prepared on 400-mesh copper grids and imaged with a transmission electron microscope (H-7100, Hitachi) operating at an acceleration voltage of 120 kV.

### Silver ion release

Concentrations of Ag^+^ released from 500 µg mL^−1^ AgNP@LA and Ag-ND@BSA in LB broth and cell culture medium were measured by using inductively coupled plasma mass spectrometry (7800 ICP-MS, Agilent). The measurements were carried out after incubation of the particles in medium for 6, 24, 48, and 72 h at 37 °C and collection of silver ions by ultrafiltration of the particle suspensions through 3 kDa cutoff membranes (Millipore). Specifically, the ^107^Ag^+^ signals were detected in helium collision mode and a calibration curve was constructed with silver nitrate standard solutions (Certipur, Millipore). To minimize silver carryover, three rinses with 5% nitric acid between runs were applied and quality control was assured once every 10 runs.

### Bacterial culture

*E. coli* (strain K12 MG1655) was obtained from Bioresource Collection and Research Center (BCRC, Taiwan). The bacteria were grown either in LB liquid broth medium (25 g L^−1^) at 37 °C for 18 h with proper aeration by orbital shaking or on solid medium plates (LB supplemented with 2% bacteriological agar) for 18 h at the same temperature. To assess the *E. coli* cell numbers, a 0.5 McFarland standard (BaSO_4_ turbidity) was prepared and used as the reference. The standard corresponded to 1.5 × 10^8^ colony-forming units (CFU) mL^−1^, which was applied (with or without dilution) throughout the entire experiments.

### Bacterial ATP levels

Antimicrobial activities were measured for *E. coli* with the cell number of 1.5 × 10^8^ CFU mL^−1^. All samples were incubated in an orbital shaking incubator (37 °C and 180 rpm) for 18 h. LB broth medium and bacteria alone served as the blank and the negative control group, respectively. The experimental groups included bacteria incubated with 5 mg mL^−1^ BSA or LA in LB broth as well as bacteria incubated with 62.5–500 µg mL^−1^ non-oxidized AgNP, oxidized AgNP, and AgNP@LA in LB broth. Additionally, time-dependent activities were assessed for bacteria incubated with 250 µg mL^−1^ AgNP@LA and Ag-ND@BSA from 1 h to 36 days. The BacTiter-Glo microbial cell assay (Promega), which measured the cell activity through a mono-oxygenation of luciferin catalyzed by firefly luciferase (an ATP-dependent enzyme), was used to assess the bacterial ATP levels. To avoid any possible interference by the nanoparticles, AgNP, AgNP@LA, and Ag-ND@BSA were removed by LB wash through centrifugal separation prior to the assays. A multimode luminometer (GloMax Discover System GM3000, Promega) measured the luminescence produced from the luciferase-catalyzed oxidative reactions.

### MIC and MBC

Bacterial suspensions with the cell number of 1.5 × 10^8^ CFU mL^−1^ were diluted to 5 × 10^5^ CFU mL^−1^ using fresh LB medium for MIC and MBC measurements. Both assays were performed according to the standard protocols recommended by the Clinical and Laboratory Standards Institute (CLSI) of USA and the European Committee on Antimicrobial Susceptibility Testing (EUCAST). In brief, a 96-well microtiter plate was first filled with *E. coli* suspensions containing 5 × 10^5^ CFU mL^−1^ per well. AgNP@LA and Ag-ND@BSA suspensions prepared by broth microdilution (500 to 7.8 µg mL^−1^) were then added to the plate. After 18 h, samples were washed with LB medium three times and re-suspended in 100 μL LB for optical density measurement (Multiskan EX, Thermo Fisher Scientific) and data were recorded for MIC analysis. Afterwards, the MBC assay was undertaken by re-culturing each sample from the MIC measurement onto fresh agar plates and observing the growth of colonies after overnight incubation.

### ROS generation

The generation of ROS in human cells was examined by using the ROS-Glo H_2_O_2_ assay (Promega), following the manufacturer’s instructions. Briefly, HFW, A549, and MCF-7 cells were pre-cultured in Dulbecco’s modified Eagle’s medium supplemented with 10% fetal bovine serum in a 96-well plate (10,000 cells/well) overnight at 37 °C and 5% CO_2_. Cells were then incubated with AgNP@LA and Ag-ND@BSA at various concentrations (62.5–500 µg mL^−1^) in culture medium for 1 h. After removal of the particles not taken up by cells through repeated pipetting and PBS wash, cells were incubated with H_2_O_2_ substrates for 6 h and were then added with the ROS-Glo detection solution for 20 min before recording the bioluminescence intensities with the luminometer.

### Cell viability

Cytotoxicity of AgNP@LA and Ag-ND@BSA was evaluated by using the cell counting kit-8 (CCK-8, Sigma-Aldrich), following the manufacturer’s instructions. In brief, cells in a 96-well plate were incubated with AgNP@LA or Ag-ND@BSA at various concentrations (62.5–500 µg mL^−1^) in culture medium for 24 h. After removal of the particles not taken up by cells through repeated pipetting and PBS washes, the CCK-8 solution was added to the wells and incubated for 30 min before measuring the absorbance of formazan at 450 nm with the multiplate reader.

### Three-in-one hydrogels

Two types of hydrogels were prepared: (1) agar composed of agarose and (2) guar gum composed of galactomannan polysaccharides extracted from guar beans. For the first type, LB-supplemented agars were mixed with Ag-ND or Ag-ND@BSA (250 µg mL^−1^ each) to form plates. Bacterial suspensions containing 1.5 × 10^8^ CFU mL^−1^ *E. coli* or 1.5 × 10^7^ CFU mL^−1^ *S. aureus* were then homogeneously spread on the agar plates and incubated overnight for observation. The antimicrobial efficacy was inspected by re-culturing scratches of the individual plates into 5 mL LB medium for 18 h. To produce guar-based wound dressing, a pre-gel solution, consisting of 0.2 wt.% agar and 1 wt.% guar gum, was first prepared and degassed under vacuum for complete bubble removal. ND@BSA or Ag-ND@BSA (250 µg mL^−1^ each) was then added into the pre-gel solution. Chemically crosslinked hydrogels formed when the particle-incorporated pre-gel solution was mixed with the ionic crosslinker, 0.6 wt.% tetraborate. The hydrogels were finally cut, reassembled, or poured into molds for shaping as desired.

## Supplementary information

Supplementary Info

## Data Availability

All the data are available on request from the corresponding author (H.C.C.).
