# Supplementary material for: Nanodiamond-supported silver nanoparticles as potent and safe antibacterial agents
Source: Sci Rep. 2019 Sep 11;9:13164. doi: 10.1038/s41598-019-49675-z (PMC6739346; doi:10.1038/s41598-019-49675-z)
Supplement: Supplementary file 1 — Supplementary Info [file 41598_2019_49675_MOESM1_ESM.pdf]

Supplementary Information

Nanodiamond-supported silver nanoparticles as potent and safe antibacterial agents

B.-M. Chang, L. Pan, H.-H. Lin, and H.-C. Chang

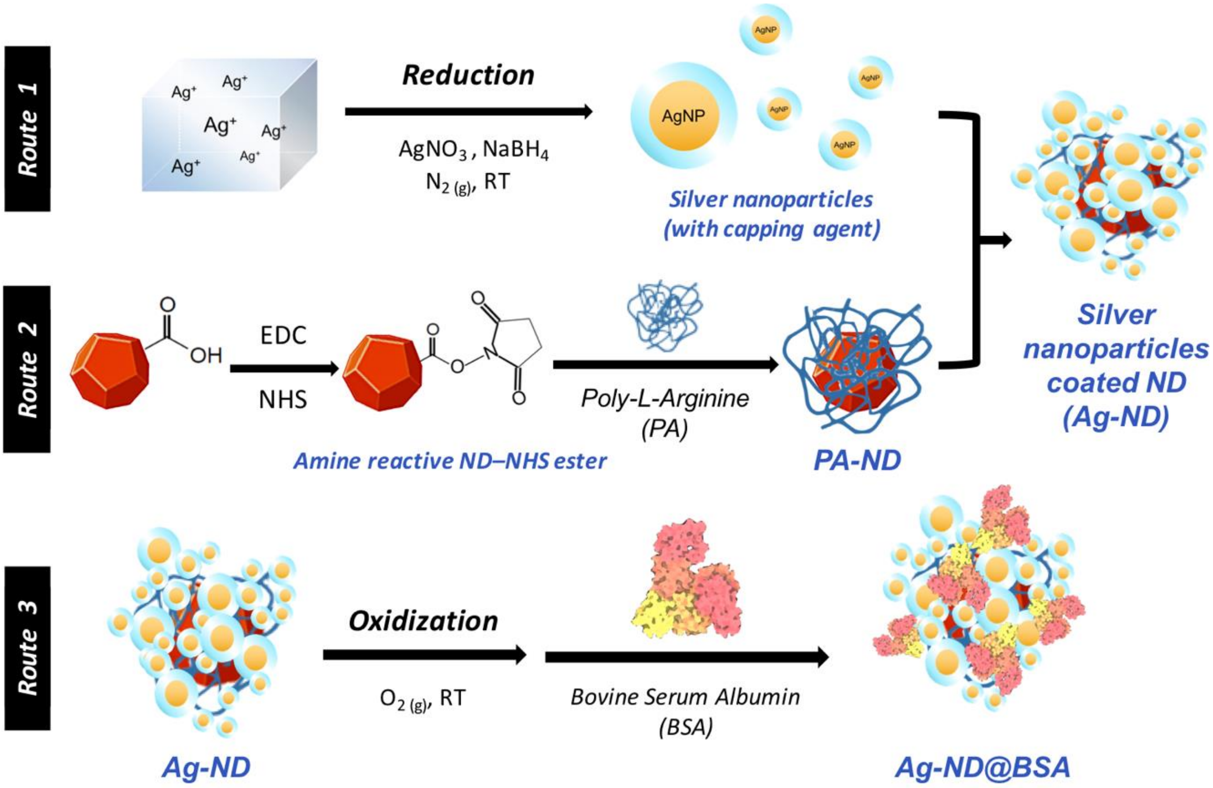

Scheme S1. Synthesis of hybrid Ag-ND and Ag-ND@BSA. Route 1: AgNP particles are grown through the chemical reduction of $AgNO_3$ by $NaBH_4$ and then stabilized by capping with citrates. Route 2: Acid-treated ND particles are surface-functionalized with PA via carbodiimide chemistry with EDC and NHS. The combination of routes 1 and 2 allows the as-grown AgNP to be anchored onto PA-ND through electrostatic attraction to form hybrid Ag-ND particles. Route 3: Ag-ND particles are oxidized in solution by oxygen gas to enhance the bactericidal activity, followed by physical adsorption of BSA on surface to achieve high dispersibility in PBS and bacterial growth medium.

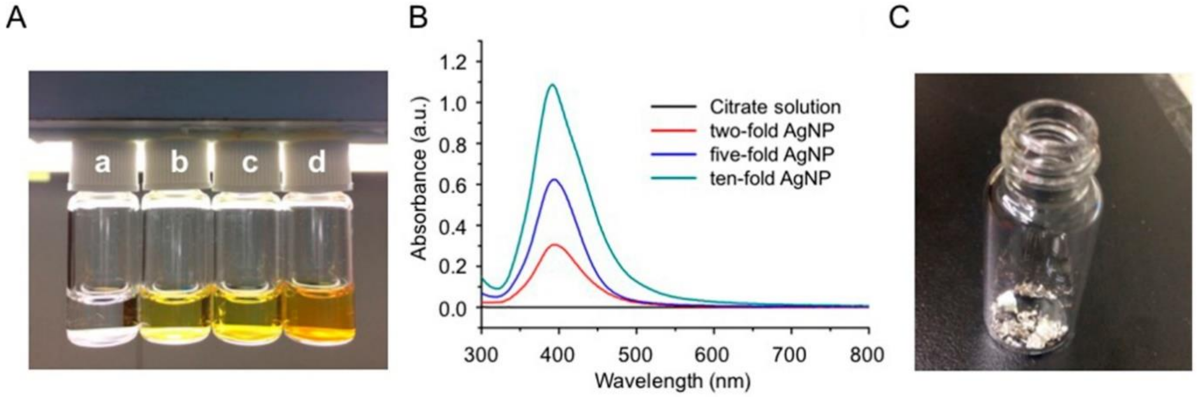

Figure S1. Characterization of AgNP. (A, B) Photograph and absorption spectra of AgNP particles dispersed in sodium citrate solution at four different concentrations, 0 mg mL^{-1} (a), 0.02 mg mL^{-1} (b), 0.05 mg mL^{-1} (c), and 0.1 mg mL^{-1} (d) in panel A. (C) Photograph of dry AgNP powders.

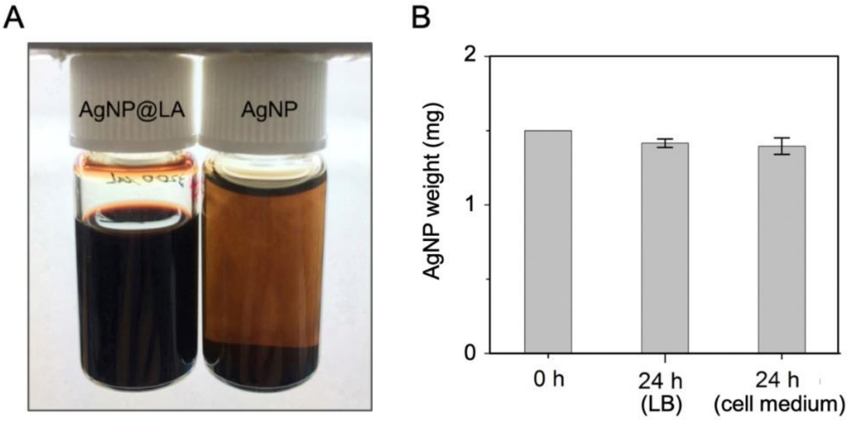

Figure S2. Identification and quantification of AgNP suspension ability. (A) Suspensions of AgNP with and without coating with LA. Aggregation and precipitation of AgNP particles without LA coating readily occurred in LB broth (right) within 15 min. The concentrations of the particles in both suspensions exceeded 1 mg mL^{-1}. (B) Quantification of AgNP precipitates in LB and cell medium at the particle concentration of 500 μg mL^{-1} after incubation for 24 h. The volumes of both LB and cell medium used in the measurements were 3 mL.

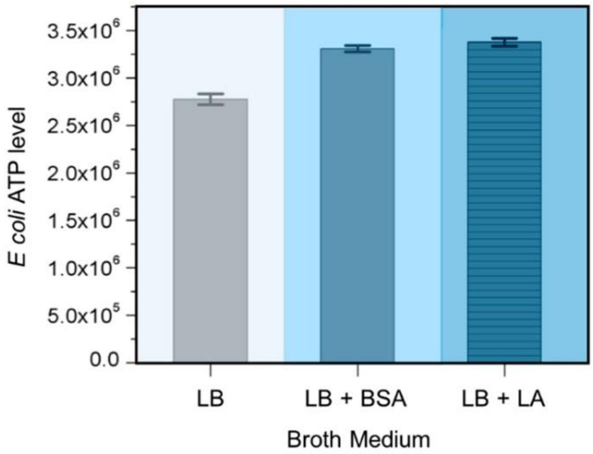

Figure S3. Antimicrobial activities of BSA and LA against *E. coli*. The experiments, conducted with *E. coli* incubated with 5 mg mL^{-1} BSA and 5 mg mL^{-1} LA for 18 h, served as controls to assess the antimicrobial activities of AgNP@LA and Ag-ND@BSA.

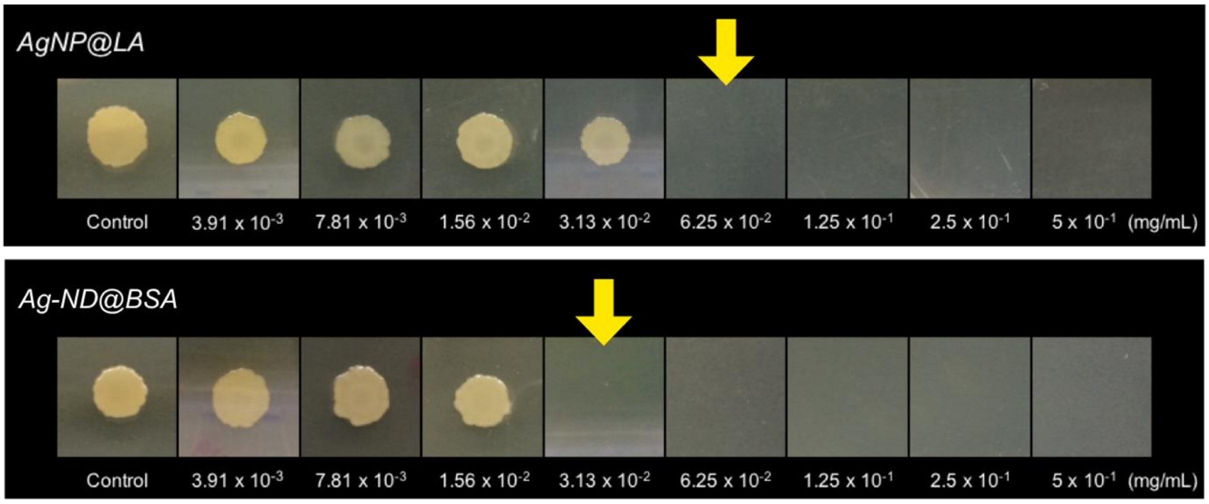

Figure S4. Minimum bactericidal concentration (MBC) assays of AgNP@LA and Ag-ND@BSA. In the assays, *E. coli* was first treated with AgNP@LA or Ag-ND@BSA at different concentrations and subsequently cultured on antibiotic-free agar plates. The lowest concentration with no visible growth of *E. coli* was taken as the MBC (indicated by arrows).

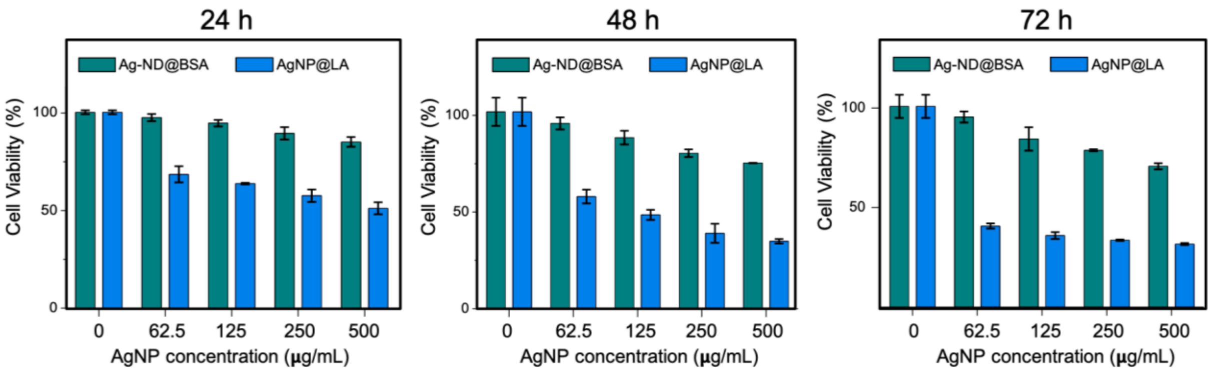

Figure S5. Cell viability of MCF-7 treated with AgNP@LA or Ag-ND@BSA. The viability assays were conducted with MCF-7 cells incubated with AgNP@LA or Ag-ND@BSA at five different concentrations for 24, 48, and 72 h.
